# Atypical cardiovocal syndrome associated with right aortic arch and Kommerell diverticulum

**DOI:** 10.4102/sajr.v30i1.3382

**Published:** 2026-03-12

**Authors:** Chetna Mishra, Vikas Yadav, Rama Anand, Moazzam Mojahid

**Affiliations:** 1 Department of Radiology, Lady Hardinge Medical College, Delhi University, New Delhi, India; 2 Department of Otorhinolaryngology, Lady Hardinge Medical College, Delhi University, New Delhi, India

**Keywords:** cardiovocal syndrome, Ortner syndrome, vocal cord palsy, right aortic arch, aortic arch anomalies, Kommerell diverticulum, recurrent laryngeal nerve, CT angiography

## Abstract

Vocal cord palsy (VCP) can be caused by laryngeal and extra-laryngeal pathologies affecting the recurrent laryngeal nerves (RLNs). Many cases of left VCP related to cardiovascular pathologies have been documented. However, right VCP associated with cardiovocal (Ortner) syndrome is an atypical and rare presentation. A case of right VCP caused by a right aortic arch (RAA) and Kommerell diverticulum (KD), diagnosed on CT angiography, and its association with the variant course of the right RLN, is described.

**Contribution:**

This case report highlights the rarity of cardiovocal syndrome causing a right VCP and the association of a RAA with the variant course of the right RLN hooking around the RAA in place of the right subclavian artery.

## Introduction

Vocal cord palsy (VCP) can be associated with laryngeal pathologies like laryngeal infections, neoplasms or trauma. The extra laryngeal causes of VCP include a wide spectrum of diseases of the central nervous system and pathologies along the course of recurrent laryngeal nerves (RLNs) in the mediastinum.^1^ Cardiovocal syndrome, also known as Ortner syndrome, was initially described by Norbert Ortner in 1897 for hoarseness of voice in patients with severe mitral stenosis with a dilated left atrium responsible for compression injury of the left RLN.^1,2^ Subsequently, other cardiovascular conditions, both congenital and acquired, were also reported to be associated with VCP and included in Ortner syndrome.

Many cases of left VCP related to cardiovascular pathologies have been documented in the literature; however, a right VCP associated with cardiovocal syndrome is extremely rare. This report describes one such case of right VCP caused by a right aortic arch (RAA) with a Kommerell diverticulum (KD), diagnosed on CT angiography (CTA).

## Patient presentation

A 20-year-old female presented with progressive hoarseness of voice over 4–5 years, which had worsened in the last 3 months. She had no symptoms of dysphagia or dyspnoea. There was no history of cough, coryza, loss of weight, choking episodes or tuberculosis contact. History of any vocal abuse or previous surgery was absent. On clinical examination, there was no palpable mass or lymph node in the neck region. Fiberoptic indirect laryngoscopy revealed a paramedian position and restricted movement of the right vocal cord with a phonatory gap, suggesting right VCP. Subjective voice analysis showed low pitch and tone, voice quality was breathy and decreased maximum phonation duration was observed for sustained vowels.

**FIGURE 1:**
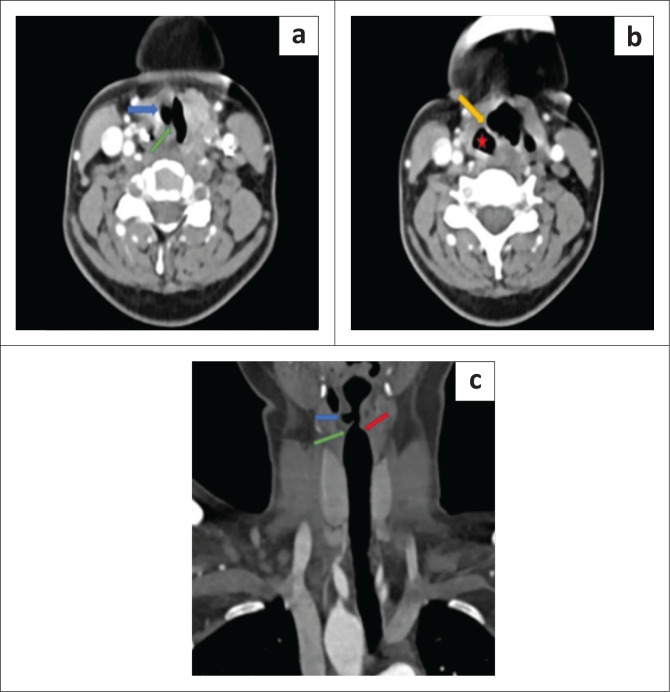
(a, b) Axial contrast-enhanced computed tomography (CECT) neck demonstrates dilatation of the right laryngeal ventricle (blue arrow), relative medial position of the right vocal cord (VC) (green arrow), dilatation of the right pyriform sinus (red star) and medial rotation of the thickened right aryepiglottic fold (yellow arrow), consistent with right vocal cord palsy. (c) Reformatted coronal CECT neck shows dilatation of the right laryngeal ventricle (blue arrow), increased concavity of the angle between the undersurface of the right VC and the right lateral wall of the larynx representing loss of the right subglottic arch (green arrow). Normal subglottic arch on the left side (red arrow).

On imaging work-up, the chest radiograph revealed a RAA. Contrast-enhanced computed tomography (CECT) of the neck demonstrated thickening and medial rotation of right aryepiglottic fold, an enlarged right pyriform sinus, dilatation of the right laryngeal ventricle and anteromedial displacement of the right arytenoid with a relatively medial position of the posterior margin of the right vocal cord, consistent with right VCP (Figure 1). No abnormal enhancement or mass was seen in the neck.

**FIGURE 2:**
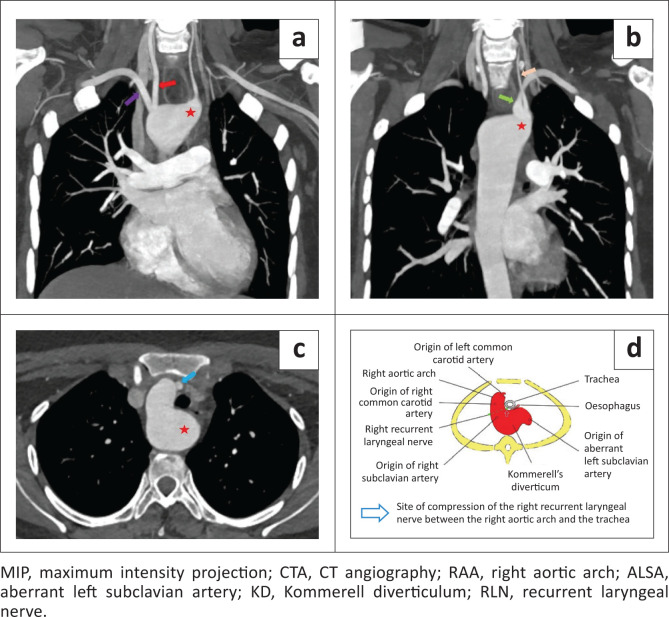
(a, b) Reformatted MIP coronal CTA images show a RAA and the origin of the right common carotid artery (red arrow) and right subclavian artery (purple arrow); The ALSA (green arrow) is arising as the fourth branch with an abnormal outpouching of the aorta at its origin, representing a KD (red star). (c) Axial CTA image reveals the KD (red star) causing anterior displacement of the trachea and oesophagus. The blue arrow demarcates the left common carotid artery just after its origin. (d) Diagrammatic illustration of the RAA with the ALSA arising from the KD and the expected site of compression of the right RLN, with a variant course, between the RAA and trachea.

**FIGURE 3:**
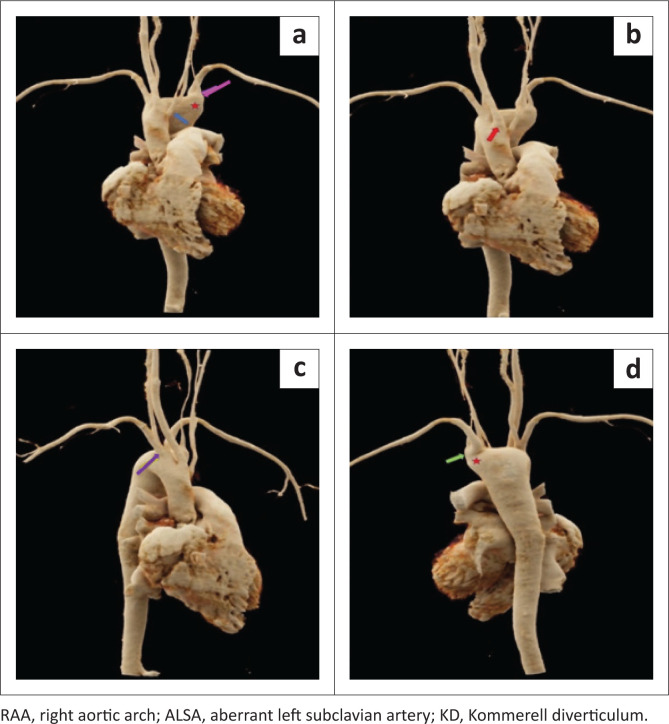
(a, b, c, d) 3D volume-rendered CT angiography of the thoracic aorta demonstrates a RAA with the left common carotid artery (blue arrow) originating as the first branch, followed by the right common carotid artery (red arrow) and the right subclavian artery (purple arrow); The ALSA (green arrow) originates from the KD (red star) as last branch. The left vertebral artery (pink arrow) is also seen arising from the KD.

CTA of the thoracic aorta performed for the evaluation of an aortic arch anomaly revealed a RAA. There was an abnormal out-pouching from the aorta representing a KD, measuring 3 cm in diameter at its origin, and the left subclavian artery was seen originating from the KD. The first branch to originate from the RAA was the left common carotid artery followed by the right common carotid artery and right subclavian artery (RSA) (Figure 2 and Figure 3). An aberrant left subclavian artery (ALSA) was seen arising as a fourth branch and revealed a retrotracheal and retro-oesophageal course. Mass effect was noted with anterior displacement of the trachea and oesophagus, secondary to the KD (Figure 2). Additional findings of a midline descending thoracic aorta, small calibre left common carotid artery with a low bifurcation and direct origin of the left vertebral artery from the KD were present (Figure 3). No other concomitant cardiac abnormality was seen.

A variant course of the right RLN hooking around the RAA was suspected, and the diagnosis of a right VCP palsy secondary to compression of the right RLN related to a RAA and KD was inferred based on imaging and anatomical correlation. The patient was referred to a cardiovascular surgeon but declined surgical intervention and was advised regular clinical and yearly imaging follow-up. The patient’s symptoms remained stable at the 6-month follow-up.

## Discussion

Cardiovocal syndrome associated with left VCP has been widely described in the literature resulting from compression of the left RLN at the aortopulmonary window. However, cardiovocal syndrome associated with a right VCP is atypical, and only a few cases have been documented.^1,3,4,5^

**FIGURE 4:**
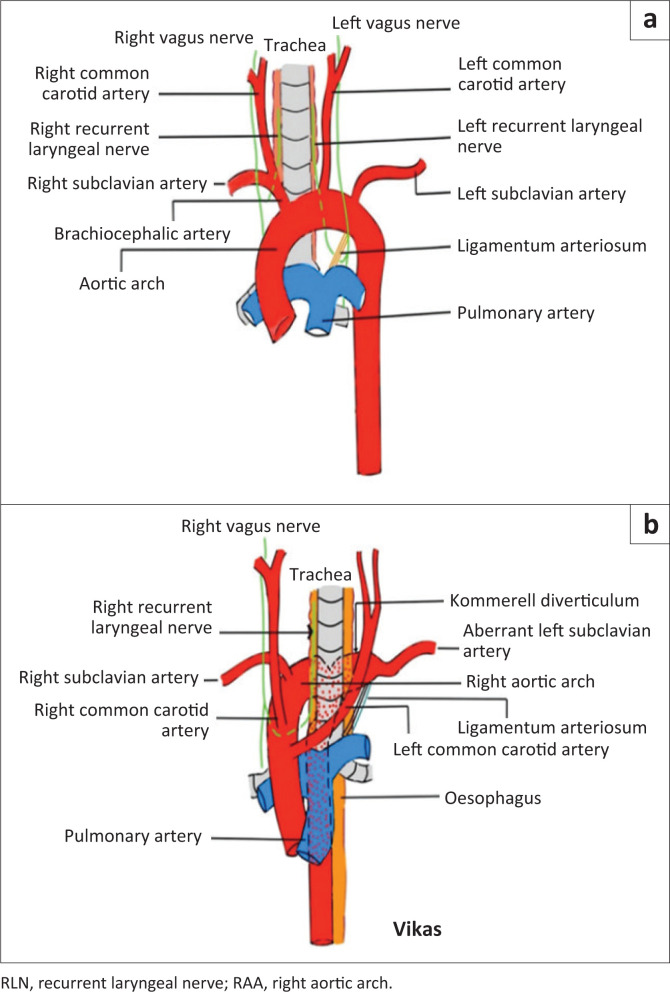
(a) Diagrammatic illustration of the normal course of the recurrent laryngeal nerves. (b) Diagrammatic illustration of a variant course of the right RLN in the presence of a RAA. In its variant course, the right RLN originates in the thorax, hooks around the RAA instead of the right subclavian artery and ascends into the tracheo-esophageal groove. This variant course of the right RLN makes it susceptible to compression between the RAA and the trachea.

The left RLN is more susceptible to injury than the right RLN because of its relatively long course, and proximity to the mediastinum, lymph nodes and aortic arch.^6^ The left RLN emerges from the left vagus nerve at the level of aortic arch, hooks back posteriorly and ascends through the superior mediastinum to reach the tracheo-esophageal groove.^2,3^ The right RLN arises from the right vagus nerve at the root of the neck and after crossing the first part of the RSA, loops back to follow a course between the trachea and the oesophagus^4,5^ (Figure 4).

On review of the literature, only a single case of right VCP associated with an aneurysm of the brachiocephalic trunk and RSA was noted.^7^ The remaining cases were associated with a RAA with or without an aortic diverticulum.^1,3,4,5^ The anatomical variant of the right RLN originating in the thorax and hooking around the RAA instead of the RSA was suspected in these cases, making it susceptible to compression injury. Both Yan et al. and Bhatnagar et al. also reported the same unusual course of the right RLN associated with the RAA in cadaveric studies.^8,9^ Although the current case lacks direct visualisation or electrophysiological confirmation, the imaging findings and literature on cadaveric studies support the presence of a variant course of right RLN hooking around the RAA, and making it susceptible to compression between the RAA and the trachea, accounting for the patient’s symptoms.

A RAA is generally asymptomatic with an incidence of 0.1% in the general population, resulting from abnormal involution of left fourth branchial arch artery during embryonic development.^10^ A RAA with an ALSA originating from a KD, as was seen in this case, is the most common variant.^10,11^ In this variant, a segment of the left dorsal aorta persists as an out-pouching from the RAA, also known as a KD, which typically has an oblique retro-oesophageal course and gives rise to the ALSA. The left ductus arteriosus or left ligamentum arteriosum forms the vascular ring in these cases, which can lead to airway or oesophageal compression.^11^ These patients are usually asymptomatic but sometimes may present with symptoms of dysphagia, dyspnoea and cough because of compression of the trachea or oesophagus, depending on the tightness of the vascular ring.^11^ However, no such symptoms were present in the current case, where the presenting symptom was hoarseness. The acute angulation of the RAA with the trachea because of the midline position of descending aorta and the mass effect by the KD, probably contributed to the compression of the right RLN between the RAA and trachea, rather than the constriction by a vascular ring.

Vocal cord palsy is usually diagnosed on clinical and laryngoscopic examination. Imaging is required to determine the underlying cause or to evaluate for suspected pathology causing the VCP. CT is the ideal modality to evaluate the larynx and is preferred to exclude distal vagal neuropathy involving the RLN. MRI is preferred if central or proximal vagal neuropathy is suspected, to localise the lesion at the brain stem or at the skull base.

Treatment of RAA with a KD depends on the severity of patient symptoms, anatomy, size of the KD, presence of associated thoracic aneurysm and complications like aortic dissection or rupture. Surgery is recommended if the KD diameter is at least 3 cm at the origin, the total aortic diameter is at least 5 cm at the level of KD or any increase in size of the diameter more than 0.5 cm per year. Open surgery with division of the ligamentum arteriosum with excision and oversewing of the KD is performed in early cases, while excision of the aneurysmal segment with aortic reconstruction and graft interposition is preferred in complex cases with a large KD. Endovascular treatment with total endovascular aortic repair using stent grafts or hybrid repair like carotid to subclavian artery bypass followed by endovascular treatment, is less invasive than open surgery; however, the choice is dependent on clinical situations and anatomical factors.^12^

A significant limitation of this report is the lack of long-term follow-up data, as the patient declined surgical intervention and was followed up for only 6 months, during which time the symptoms remained stable.

## Conclusion

A RAA with a KD associated with a right VCP should be considered as a cause of cardiovocal syndrome. This condition merits timely diagnosis and management, because of the associated morbidity from compression of the mediastinal structures and mortality from aortic dissection or rupture.

## References

[1] Rizvi MM, Singh RB, Jain A, Sarkar A. Asymptomatic aortic aneurysm causing right vocal cord palsy and hoarseness: A rare presentation. Anesth Essays Res. 2014;8(3):397–400. 10.4103/0259-1162.143157 25886343 PMC4258979

[2] Kheok SW, Salkade PR, Bangaragiri A, Koh NSY, Chen RC. Cardiovascular hoarseness (Ortner’s syndrome): A pictorial review. Curr Probl Diagn Radiol. 2021;50(5):749–754. 10.1067/j.cpradiol.2020.09.015 33036813

[3] Hazarika P, Punnoose SE, Arora S, Diesh RS, Itgampalli RK, Singh R. Hoarseness due to right vocal cord paralysis associated with aortic diverticulum from right aortic arch – A rare and unusual vascular etiology of right vocal cord paralysis. Int J Otolaryngol Head Neck Surg. 2015;4(2):99. 10.4236/ijohns.2015.42018

[4] Karaoke O, Birkent H, Aydin U, Yildizoglu U, Ilica T, Asik B. Recurrent vocal cord paralysis associated with right aortic arch. KBB-Forum Electr J Otolaryngol Head Neck Surg. 2013;12(4):1–4.

[5] Kumar P, Singh A, Chandrashekhara SH. Kommerell’s diverticulum: Rare cause of unilateral vocal cord palsy. BMJ Case Rep CP. 2019;12:e227682. 10.1136/bcr-2018-227682 PMC6887411 31767601

[6] Titche LL. Causes of recurrent laryngeal nerve paralysis. Arch Otolaryngol. 1976;102(5):259–261. 10.1001/archotol.1976.00780100045002 1267716

[7] Klee K, Eick C, Witlandt R, Gawaz M, Didczuneit-Sandhop B. Unilateral recurrent nerve palsy and cardiovascular disease – Ortner’s syndrome. J Cardiol Cases. 2016;15(3):88–90. 10.1016/j.jccase.2016.10.018 30279747 PMC6135008

[8] Yan J, Kanazawa J, Numata N, Hitomi J. The right-sided aortic arch with unusual course of bilateral recurrent laryngeal nerves: A report of rare variations. Surg Radiol Anat. 2017;39(2):223–228. 10.1007/s00276-016-1717-7 27341832

[9] Bhatnagar KP, Wagner CE, Kuwabara N, Nettleton GS, Campbell FR. Right-sided aorta: A cadaver report and brief discussion of human aortic arch anomalies. Ann Anat. 2000;182(6):559–562. 10.1016/S0940-9602(00)80104-1 11125807

[10] Hanneman K, Newman B, Chan F. Congenital variants and anomalies of the aortic arch. Radiographics. 2017;37(1):32–51. 10.1148/rg.2017160033 27860551

[11] Priya S, Thomas R, Nagpal P, Sharma A, Steigner M. Congenital anomalies of aortic arch. Cardiovasc Diagn Ther. 2018;8(Suppl. 1):S26–S44. 10.21037/cdt.2017.10.15 29850417 PMC5949580

[12] Saran N, Dearani J, Said S, et al. Vascular rings in adults: Outcome of surgical management. Ann Thorac Surg. 2019;108(4):1217–1227. 10.1016/j.athoracsur.2019.04.097 31229482

